# 3-(4-Amino­phen­yl)-5-(4-meth­oxy­phen­yl)-4,5-di­hydro-1*H*-pyrazole-1-carbo­thio­amide

**DOI:** 10.1107/S1600536813018096

**Published:** 2013-07-10

**Authors:** Thitipone Suwunwong, Suchada Chantrapromma, C. S. Chidan Kumar, Hoong-Kun Fun

**Affiliations:** a Department of Chemistry, Faculty of Science, Prince of Songkla University, Hat-Yai, Songkhla 90112, Thailand; b X-ray Crystallography Unit, School of Physics, Universiti Sains Malaysia, 11800 USM, Penang, Malaysia; c Department of Pharmaceutical Chemistry, College of Pharmacy, King Saud University, PO Box 2457, Riyadh 11451, Saudi Arabia

## Abstract

In the mol­ecule of title pyrazoline derivative, C_17_H_18_N_4_OS, the pyrazole ring adopts an envelope conformation with the flap atom, which bears the meth­oxy­phenyl substituent, displaced by 0.0750 (12) Å from the plane through the other ring atoms. The two substituted benzene rings make a dihedral angle of 70.59 (6)°. The meth­oxy group is twisted slightly with respect to the attached benzene ring [C_meth­yl_—O—C—C torsion angle = −8.84 (15)°]. An intra­molecular N—H⋯N hydrogen bond occurs. In the crystal, the pyrazoline mol­ecules are linked by N—H⋯O and N—H⋯S hydrogen bonds into zigzag layers parallel to the *bc* plane and stacked along the *a* axis by π–π inter­actions with centroid–centroid distances of 3.4690 (7) and 3.5792 (7) Å. C—H⋯π inter­actions are also present.

## Related literature

For bond-length data, see: Allen *et al.* (1987 ▶). For hydrogen-bond motifs, see: Bernstein *et al.* (1995 ▶). For puckering parameters, see: Cremer & Pople (1975 ▶). For related structures, see: Fun *et al.* (2011 ▶); Quah *et al.* (2013 ▶). For background to and applications of pyrazoline derivatives, see: Gong *et al.* (2010 ▶); Husain *et al.* (2008 ▶); Khode *et al.* (2009 ▶); Lv *et al.* (2011 ▶); Sakthinathan *et al.* (2012 ▶); Shaharyar *et al.* (2010 ▶); Shoman *et al.* (2009 ▶). For the stability of the temperature controller, see: Cosier & Glazer (1986 ▶).
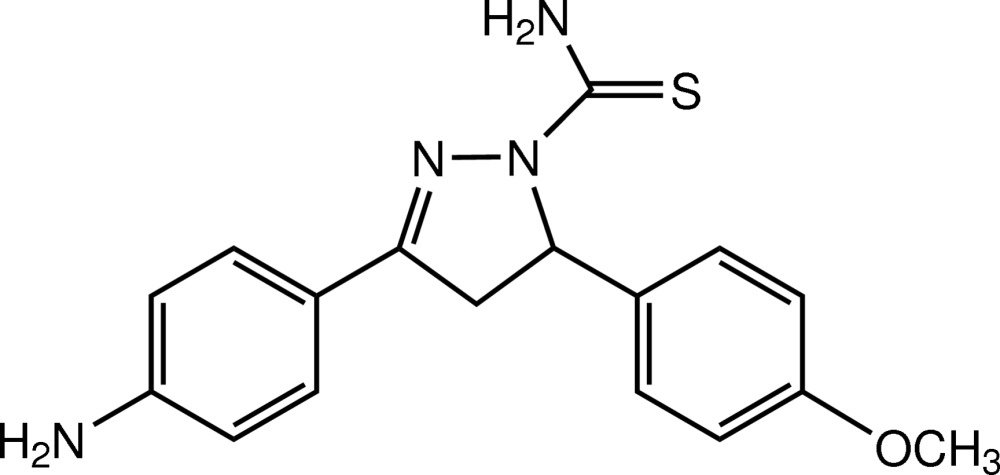


## Experimental

### 

#### Crystal data

C_17_H_18_N_4_OS
*M* *_r_* = 326.42
Monoclinic, 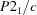

*a* = 8.0052 (2) Å
*b* = 17.3439 (5) Å
*c* = 12.4588 (3) Å
β = 114.789 (1)°
*V* = 1570.41 (7) Å^3^
*Z* = 4
Mo *K*α radiation
μ = 0.22 mm^−1^
*T* = 100 K
0.57 × 0.39 × 0.29 mm

#### Data collection

Bruker APEXII CCD area detector diffractometer
Absorption correction: multi-scan (*SADABS*; Bruker, 2009 ▶) *T*_min_ = 0.886, *T*_max_ = 0.940
23819 measured reflections
4571 independent reflections
4045 reflections with *I* > 2σ(*I*)
*R*_int_ = 0.034

#### Refinement

*R*[*F* ^2^ > 2σ(*F* ^2^)] = 0.036
*wR*(*F* ^2^) = 0.104
*S* = 1.05
4571 reflections
225 parameters
H atoms treated by a mixture of independent and constrained refinement
Δρ_max_ = 0.43 e Å^−3^
Δρ_min_ = −0.26 e Å^−3^

### 

Data collection: *APEX2* (Bruker, 2009 ▶); cell refinement: *SAINT* (Bruker, 2009 ▶); data reduction: *SAINT*; program(s) used to solve structure: *SHELXTL* (Sheldrick, 2008 ▶); program(s) used to refine structure: *SHELXTL*; molecular graphics: *SHELXTL*; software used to prepare material for publication: *SHELXTL*, *PLATON* (Spek, 2009 ▶) and *publCIF* (Westrip, 2010 ▶).

## Supplementary Material

Crystal structure: contains datablock(s) global, I. DOI: 10.1107/S1600536813018096/rz5075sup1.cif

Structure factors: contains datablock(s) I. DOI: 10.1107/S1600536813018096/rz5075Isup2.hkl

Supplementary material file. DOI: 10.1107/S1600536813018096/rz5075Isup3.cml

Additional supplementary materials:  crystallographic information; 3D view; checkCIF report

## Figures and Tables

**Table 1:** Hydrogen-bond geometry (Å, °) *Cg*2 is the centroid of the C1–C6 ring.

*D*—H⋯*A*	*D*—H	H⋯*A*	*D*⋯*A*	*D*—H⋯*A*
N3—H1*N*3⋯S1^i^	0.866 (17)	2.664 (17)	3.4559 (12)	152.5 (15)
N3—H2*N*3⋯S1^ii^	0.84 (2)	2.60 (2)	3.4142 (12)	164.2 (18)
N4—H2*N*4⋯N1	0.881 (17)	2.209 (16)	2.6093 (15)	107.2 (13)
N4—H2*N*4⋯O1^ii^	0.881 (17)	2.567 (17)	3.3022 (14)	141.5 (13)
C8—H8*A*⋯*Cg*2^i^	0.99	2.66	3.4159 (13)	133

Symmetry codes: (i) 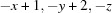; (ii) 
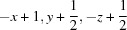
.

**Fig. 1.**
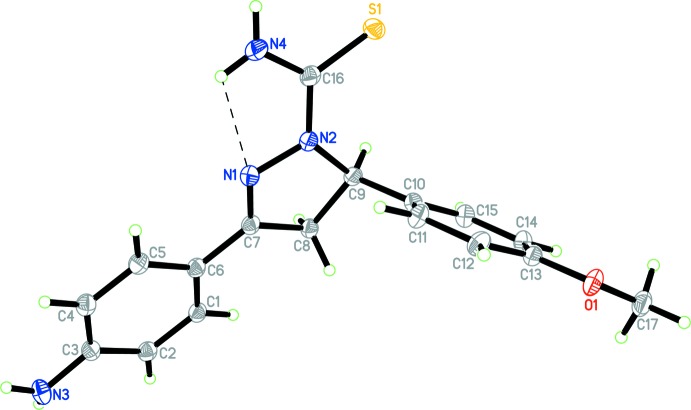
The molecular structure of the title compound, showing 50% probability displacement ellipsoids and the atom-numbering scheme. Intramolecular N—H···N hydrogen bond is shown as a dashed line.

**Fig. 2.**
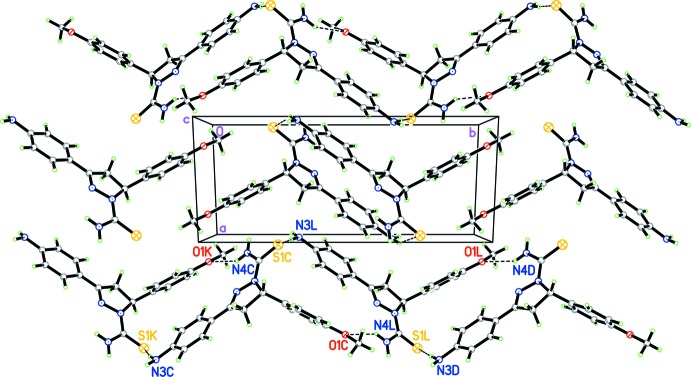
Crystal packing of the title compound viewed along the c axis. Hydrogen bonds are shown as dashed lines.

## supplementary crystallographic information

### Comment

Pyrazolines are five-membered heterocyclic compounds having three carbon atoms and two adjacent nitrogen atoms within the pyrazoline ring. Numerous pyrazolines have been found to possess considerable biological activities with several prominent effects, such as antimicrobial (Sakthinathan *et al.*, 2012), antiamoebic (Husain *et al.*, 2008), anti-inflammatory (Shoman *et al.*, 2009), analgesic (Khode *et al.*, 2009) and anticancer (Lv *et al.*, 2011; Shaharyar *et al.*, 2010) activities, as well as optical properties (Gong *et al.*, 2010). Owing to these interesting properties of pyrazolines and our on-going research on fluorescence and biologically active compounds, the title pyrazoline derivative (I) was synthesized by cyclization of the chalcone derivative with thiosemicarbazide. Herein the crystal structure of (I) is reported.

In the molecule of (I), C_17_H_18_N_4_OS, the pyrazole ring is in an envelope conformation (pucker atom at C9 with deviation of 0.0750 (12) Å) with puckering parameter Q = 0.1186 (12) Å and φ = 71.2 (5)° (Cremer & Pople, 1975). The mean plane through pyrazole ring makes the dihedral angles of 5.75 (6) and 73.76 (6)° with 4-aminophenyl and 4-methoxyphenyl rings, respectively, whereas the dihedral angle between the two substituted phenyl rings is 70.59 (6)°. The methoxy group is slightly twisted from its attached benzene ring with the torsion angle C17–O1–C13–C14 = -8.84 (15)°. The carbothioamide unit is also twisted from pyrazole ring as indicated by the torsion angles N21—N2–C16–N4 = 9.07 (15)° and N1–N2–C16–S2 = -171.50 (8)°. An intramolecular N4—H2N4···N1 hydrogen bond generates an S(5) ring motif (Fig. 1; Bernstein *et al.*, 1995). Bond distances in (I) are in normal ranges (Allen *et al.*, 1987) and comparable with those observed in related structures (Fun *et al.*, 2011; Quah *et al.*, 2013).

In the crystal packing (Fig. 2), the molecules are linked in a zigzag fashion by N_amino_—H···S and N_thioamide_—H···O intermolcular interactions (Table 1) into a two dimensional network parallel to the *bc* plane which further stacks along the *a*-axis by π···π interactions with centroid..centroid distances of *Cg*_1_···*Cg*_2_^ii^ = 3.5792 (7) Å and *Cg*_2_···*Cg*_2_^v^ = 3.4690 (7) Å [symmetry code (v) = -*x*, 2 - *y*, -*z* and *Cg*_1_ is the centroid of N1/N2/C7–C9 ring]. C—H···π interactions (Table 1) are also present.

### Experimental

The title compound was synthesized by dissolving (*E*)-1-(4-aminophenyl)-3-(4-methoxyphenyl)prop-2-en-1-one (0.25 g, 1.0 mmol) in a solution of KOH (0.11 g, 2.0 mmol) in ethanol (20 ml). An excess thiosemicarbazide (0.18 g, 2.0 mmol) in ethanol (20 ml) was then added, and the reaction mixture was vigorously stirred and refluxed for 4 h. The brown solid of the title compound obtained after cooling was filtered off under vacuum. Brown block-shaped single crystals of the title compound suitable for *X*-ray structure determination were recrystallized from C_2_H_5_OH by slow evaporation of the solvent at room temperature after several days. M. p. 479–480 K.

### Refinement

Amino and thioamide H atoms were located in a difference Fourier map and refined isotropically. The remaining H atoms were positioned geometrically and allowed to ride on their parent atoms, with d(C—H) = 0.95 Å for aromatic, 1.00 Å for CH, 0.99 Å for CH_2_ and 0.98 for CH_3_ atoms. The *U*_iso_ values were constrained to be 1.5*U*_eq_ of the carrier atom for methyl H atoms and 1.2*U*_eq_ for the remaining H atoms. A rotating group model was used for the methyl group.

### Crystal data

**Table:**

C_17_H_18_N_4_OS	*F*(000) = 688
*M**_r_* = 326.42	*D*_x_ = 1.381 Mg m^−^^3^
Monoclinic, *P*2_1_/*c*	Melting point = 479–480 K
Hall symbol: -P 2ybc	Mo *K*α radiation, λ = 0.71073 Å
*a* = 8.0052 (2) Å	Cell parameters from 4571 reflections
*b* = 17.3439 (5) Å	θ = 2.2–30.0°
*c* = 12.4588 (3) Å	µ = 0.22 mm^−^^1^
β = 114.789 (1)°	*T* = 100 K
*V* = 1570.41 (7) Å^3^	Block, brown
*Z* = 4	0.57 × 0.39 × 0.29 mm

### Data collection

**Table:**

Bruker APEXII CCD area detector diffractometer	4571 independent reflections
Radiation source: sealed tube	4045 reflections with *I* > 2σ(*I*)
Graphite monochromator	*R*_int_ = 0.034
φ and ω scans	θ_max_ = 30.0°, θ_min_ = 2.2°
Absorption correction: multi-scan (*SADABS*; Bruker, 2009)	*h* = −11→11
*T*_min_ = 0.886, *T*_max_ = 0.940	*k* = −24→22
23819 measured reflections	*l* = −17→17

### Refinement

**Table:**

Refinement on *F*^2^	Primary atom site location: structure-invariant direct methods
Least-squares matrix: full	Secondary atom site location: difference Fourier map
*R*[*F*^2^ > 2σ(*F*^2^)] = 0.036	Hydrogen site location: inferred from neighbouring sites
*wR*(*F*^2^) = 0.104	H atoms treated by a mixture of independent and constrained refinement
*S* = 1.05	*w* = 1/[σ^2^(*F*_o_^2^) + (0.0561*P*)^2^ + 0.5572*P*] where *P* = (*F*_o_^2^ + 2*F*_c_^2^)/3
4571 reflections	(Δ/σ)_max_ = 0.002
225 parameters	Δρ_max_ = 0.43 e Å^−^^3^
0 restraints	Δρ_min_ = −0.26 e Å^−^^3^

### Special details

**Table:**

Experimental. The crystal was placed in the cold stream of an Oxford Cryosystems Cobra open-flow nitrogen cryostat (Cosier & Glazer, 1986) operating at 100.0 (1) K.
Geometry. All e.s.d.'s (except the e.s.d. in the dihedral angle between two l.s. planes) are estimated using the full covariance matrix. The cell e.s.d.'s are taken into account individually in the estimation of e.s.d.'s in distances, angles and torsion angles; correlations between e.s.d.'s in cell parameters are only used when they are defined by crystal symmetry. An approximate (isotropic) treatment of cell e.s.d.'s is used for estimating e.s.d.'s involving l.s. planes.
Refinement. Refinement of *F*^2^ against ALL reflections. The weighted *R*-factor *wR* and goodness of fit *S* are based on *F*^2^, conventional *R*-factors *R* are based on *F*, with *F* set to zero for negative *F*^2^. The threshold expression of *F*^2^ > σ(*F*^2^) is used only for calculating *R*-factors(gt) *etc*. and is not relevant to the choice of reflections for refinement. *R*-factors based on *F*^2^ are statistically about twice as large as those based on *F*, and *R*- factors based on ALL data will be even larger.

### Fractional atomic coordinates and isotropic or equivalent isotropic displacement parameters (Å^2^)

**Table:**

	*x*	*y*	*z*	*U*_iso_*/*U*_eq_	
S1	0.96189 (4)	0.737361 (16)	0.23440 (3)	0.02103 (9)	
O1	0.20880 (12)	0.50083 (5)	0.02032 (7)	0.02307 (18)	
N1	0.56007 (13)	0.88586 (5)	0.17966 (8)	0.01819 (18)	
N2	0.65454 (13)	0.82048 (5)	0.16720 (8)	0.01788 (18)	
N3	−0.00793 (15)	1.16984 (6)	−0.00281 (10)	0.0223 (2)	
N4	0.88104 (15)	0.85193 (6)	0.34723 (9)	0.0236 (2)	
C1	0.20776 (14)	0.99640 (6)	−0.05879 (10)	0.0176 (2)	
H1A	0.2020	0.9666	−0.1242	0.021*	
C2	0.10060 (15)	1.06212 (6)	−0.07863 (10)	0.0183 (2)	
H2A	0.0237	1.0772	−0.1573	0.022*	
C3	0.10467 (15)	1.10666 (6)	0.01667 (10)	0.0181 (2)	
C4	0.22393 (15)	1.08391 (6)	0.13240 (10)	0.0191 (2)	
H4A	0.2304	1.1138	0.1979	0.023*	
C5	0.33141 (15)	1.01856 (6)	0.15128 (10)	0.0182 (2)	
H5A	0.4112	1.0042	0.2298	0.022*	
C6	0.32452 (14)	0.97303 (6)	0.05611 (10)	0.0168 (2)	
C7	0.43893 (14)	0.90464 (6)	0.07541 (9)	0.0165 (2)	
C8	0.43688 (14)	0.85248 (6)	−0.02203 (9)	0.0169 (2)	
H8A	0.4806	0.8799	−0.0751	0.020*	
H8B	0.3119	0.8319	−0.0693	0.020*	
C9	0.57125 (14)	0.78746 (6)	0.04662 (9)	0.0167 (2)	
H9A	0.6676	0.7810	0.0160	0.020*	
C10	0.47626 (14)	0.71095 (6)	0.04153 (9)	0.0163 (2)	
C11	0.41276 (16)	0.68894 (7)	0.12532 (10)	0.0202 (2)	
H11A	0.4310	0.7222	0.1898	0.024*	
C12	0.32283 (16)	0.61881 (7)	0.11590 (10)	0.0211 (2)	
H12A	0.2805	0.6045	0.1739	0.025*	
C13	0.29479 (14)	0.56940 (6)	0.02121 (10)	0.0180 (2)	
C14	0.35358 (16)	0.59149 (6)	−0.06472 (10)	0.0202 (2)	
H14A	0.3321	0.5590	−0.1306	0.024*	
C15	0.44439 (16)	0.66176 (6)	−0.05325 (10)	0.0199 (2)	
H15A	0.4855	0.6764	−0.1116	0.024*	
C16	0.82554 (15)	0.80698 (6)	0.25017 (10)	0.0185 (2)	
C17	0.15491 (17)	0.45460 (7)	−0.08438 (11)	0.0252 (2)	
H17A	0.0945	0.4075	−0.0751	0.038*	
H17B	0.2641	0.4408	−0.0970	0.038*	
H17C	0.0694	0.4839	−0.1526	0.038*	
H1N3	−0.039 (2)	1.1943 (10)	−0.0690 (15)	0.030 (4)*	
H2N3	0.007 (3)	1.1952 (11)	0.0576 (17)	0.038 (5)*	
H1N4	0.992 (3)	0.8465 (11)	0.3959 (17)	0.041 (5)*	
H2N4	0.810 (2)	0.8906 (10)	0.3477 (14)	0.031 (4)*	

### Atomic displacement parameters (Å^2^)

**Table:**

	*U*^11^	*U*^22^	*U*^33^	*U*^12^	*U*^13^	*U*^23^
S1	0.01945 (14)	0.01830 (14)	0.02342 (15)	0.00144 (9)	0.00711 (11)	0.00405 (10)
O1	0.0271 (4)	0.0168 (4)	0.0234 (4)	−0.0078 (3)	0.0088 (3)	−0.0032 (3)
N1	0.0184 (4)	0.0153 (4)	0.0204 (4)	−0.0008 (3)	0.0076 (3)	−0.0010 (3)
N2	0.0175 (4)	0.0147 (4)	0.0187 (4)	−0.0008 (3)	0.0049 (3)	−0.0012 (3)
N3	0.0288 (5)	0.0160 (4)	0.0255 (5)	0.0033 (4)	0.0146 (4)	0.0015 (4)
N4	0.0210 (5)	0.0219 (5)	0.0208 (5)	−0.0010 (4)	0.0017 (4)	−0.0021 (4)
C1	0.0182 (5)	0.0154 (5)	0.0195 (5)	−0.0025 (4)	0.0083 (4)	−0.0013 (4)
C2	0.0189 (5)	0.0164 (5)	0.0197 (5)	−0.0019 (4)	0.0082 (4)	0.0008 (4)
C3	0.0185 (5)	0.0146 (4)	0.0241 (5)	−0.0023 (4)	0.0116 (4)	0.0004 (4)
C4	0.0211 (5)	0.0180 (5)	0.0210 (5)	−0.0026 (4)	0.0115 (4)	−0.0019 (4)
C5	0.0194 (5)	0.0178 (5)	0.0183 (5)	−0.0027 (4)	0.0087 (4)	0.0002 (4)
C6	0.0172 (4)	0.0143 (4)	0.0196 (5)	−0.0024 (4)	0.0086 (4)	−0.0006 (4)
C7	0.0164 (4)	0.0149 (4)	0.0192 (5)	−0.0031 (3)	0.0086 (4)	0.0000 (4)
C8	0.0177 (4)	0.0142 (4)	0.0181 (5)	−0.0009 (3)	0.0068 (4)	−0.0001 (4)
C9	0.0163 (4)	0.0156 (4)	0.0173 (5)	−0.0016 (3)	0.0060 (4)	−0.0007 (4)
C10	0.0155 (4)	0.0139 (4)	0.0179 (5)	0.0002 (3)	0.0053 (4)	0.0003 (4)
C11	0.0228 (5)	0.0188 (5)	0.0190 (5)	−0.0045 (4)	0.0089 (4)	−0.0039 (4)
C12	0.0228 (5)	0.0223 (5)	0.0188 (5)	−0.0054 (4)	0.0094 (4)	−0.0014 (4)
C13	0.0161 (4)	0.0143 (4)	0.0204 (5)	−0.0009 (3)	0.0044 (4)	0.0004 (4)
C14	0.0239 (5)	0.0164 (5)	0.0205 (5)	−0.0010 (4)	0.0096 (4)	−0.0039 (4)
C15	0.0236 (5)	0.0171 (5)	0.0205 (5)	−0.0012 (4)	0.0108 (4)	−0.0009 (4)
C16	0.0184 (5)	0.0156 (5)	0.0192 (5)	−0.0031 (4)	0.0057 (4)	0.0028 (4)
C17	0.0284 (6)	0.0175 (5)	0.0245 (6)	−0.0053 (4)	0.0061 (5)	−0.0047 (4)

### Geometric parameters (Å, º)

**Table:**

S1—C16	1.6935 (12)	C5—H5A	0.9500
O1—C13	1.3719 (13)	C6—C7	1.4557 (15)
O1—C17	1.4349 (14)	C7—C8	1.5086 (15)
N1—C7	1.2951 (14)	C8—C9	1.5457 (15)
N1—N2	1.4072 (13)	C8—H8A	0.9900
N2—C16	1.3456 (14)	C8—H8B	0.9900
N2—C9	1.4798 (14)	C9—C10	1.5173 (14)
N3—C3	1.3744 (14)	C9—H9A	1.0000
N3—H1N3	0.866 (18)	C10—C15	1.3910 (15)
N3—H2N3	0.836 (19)	C10—C11	1.3924 (15)
N4—C16	1.3479 (15)	C11—C12	1.3931 (15)
N4—H1N4	0.85 (2)	C11—H11A	0.9500
N4—H2N4	0.882 (18)	C12—C13	1.3980 (16)
C1—C2	1.3851 (15)	C12—H12A	0.9500
C1—C6	1.4018 (15)	C13—C14	1.3918 (16)
C1—H1A	0.9500	C14—C15	1.3953 (15)
C2—C3	1.4053 (15)	C14—H14A	0.9500
C2—H2A	0.9500	C15—H15A	0.9500
C3—C4	1.4113 (16)	C17—H17A	0.9800
C4—C5	1.3822 (15)	C17—H17B	0.9800
C4—H4A	0.9500	C17—H17C	0.9800
C5—C6	1.4063 (15)		
			
C13—O1—C17	116.77 (9)	C9—C8—H8B	111.2
C7—N1—N2	107.65 (9)	H8A—C8—H8B	109.1
C16—N2—N1	118.43 (9)	N2—C9—C10	112.67 (9)
C16—N2—C9	126.36 (10)	N2—C9—C8	101.08 (8)
N1—N2—C9	112.93 (8)	C10—C9—C8	113.24 (8)
C3—N3—H1N3	117.7 (11)	N2—C9—H9A	109.8
C3—N3—H2N3	115.2 (13)	C10—C9—H9A	109.8
H1N3—N3—H2N3	118.3 (17)	C8—C9—H9A	109.8
C16—N4—H1N4	115.4 (13)	C15—C10—C11	118.26 (10)
C16—N4—H2N4	118.4 (11)	C15—C10—C9	118.83 (10)
H1N4—N4—H2N4	124.6 (16)	C11—C10—C9	122.86 (10)
C2—C1—C6	121.20 (10)	C10—C11—C12	120.97 (10)
C2—C1—H1A	119.4	C10—C11—H11A	119.5
C6—C1—H1A	119.4	C12—C11—H11A	119.5
C1—C2—C3	120.55 (10)	C11—C12—C13	120.05 (11)
C1—C2—H2A	119.7	C11—C12—H12A	120.0
C3—C2—H2A	119.7	C13—C12—H12A	120.0
N3—C3—C2	120.53 (10)	O1—C13—C14	124.25 (10)
N3—C3—C4	121.06 (10)	O1—C13—C12	116.15 (10)
C2—C3—C4	118.40 (10)	C14—C13—C12	119.60 (10)
C5—C4—C3	120.62 (10)	C13—C14—C15	119.43 (10)
C5—C4—H4A	119.7	C13—C14—H14A	120.3
C3—C4—H4A	119.7	C15—C14—H14A	120.3
C4—C5—C6	121.02 (10)	C10—C15—C14	121.67 (10)
C4—C5—H5A	119.5	C10—C15—H15A	119.2
C6—C5—H5A	119.5	C14—C15—H15A	119.2
C1—C6—C5	118.19 (10)	N2—C16—N4	115.79 (10)
C1—C6—C7	120.55 (10)	N2—C16—S1	122.17 (9)
C5—C6—C7	121.24 (10)	N4—C16—S1	122.03 (9)
N1—C7—C6	121.70 (10)	O1—C17—H17A	109.5
N1—C7—C8	114.02 (9)	O1—C17—H17B	109.5
C6—C7—C8	124.19 (9)	H17A—C17—H17B	109.5
C7—C8—C9	102.83 (8)	O1—C17—H17C	109.5
C7—C8—H8A	111.2	H17A—C17—H17C	109.5
C9—C8—H8A	111.2	H17B—C17—H17C	109.5
C7—C8—H8B	111.2		
			
C7—N1—N2—C16	155.52 (10)	N1—N2—C9—C8	12.22 (11)
C7—N1—N2—C9	−8.44 (12)	C7—C8—C9—N2	−10.76 (10)
C6—C1—C2—C3	−0.83 (16)	C7—C8—C9—C10	110.01 (9)
C1—C2—C3—N3	−176.99 (10)	N2—C9—C10—C15	−159.59 (10)
C1—C2—C3—C4	1.66 (16)	C8—C9—C10—C15	86.46 (12)
N3—C3—C4—C5	177.48 (10)	N2—C9—C10—C11	23.02 (14)
C2—C3—C4—C5	−1.17 (16)	C8—C9—C10—C11	−90.94 (12)
C3—C4—C5—C6	−0.18 (16)	C15—C10—C11—C12	1.24 (17)
C2—C1—C6—C5	−0.52 (16)	C9—C10—C11—C12	178.65 (10)
C2—C1—C6—C7	−178.81 (10)	C10—C11—C12—C13	−0.13 (17)
C4—C5—C6—C1	1.02 (16)	C17—O1—C13—C14	−8.84 (15)
C4—C5—C6—C7	179.30 (10)	C17—O1—C13—C12	171.16 (10)
N2—N1—C7—C6	−176.28 (9)	C11—C12—C13—O1	178.59 (10)
N2—N1—C7—C8	0.30 (12)	C11—C12—C13—C14	−1.41 (17)
C1—C6—C7—N1	173.53 (10)	O1—C13—C14—C15	−178.19 (10)
C5—C6—C7—N1	−4.71 (16)	C12—C13—C14—C15	1.81 (16)
C1—C6—C7—C8	−2.69 (16)	C11—C10—C15—C14	−0.84 (16)
C5—C6—C7—C8	179.07 (10)	C9—C10—C15—C14	−178.35 (10)
N1—C7—C8—C9	7.16 (12)	C13—C14—C15—C10	−0.69 (17)
C6—C7—C8—C9	−176.35 (9)	N1—N2—C16—N4	9.07 (15)
C16—N2—C9—C10	88.62 (13)	C9—N2—C16—N4	170.65 (10)
N1—N2—C9—C10	−108.95 (10)	N1—N2—C16—S1	−171.50 (8)
C16—N2—C9—C8	−150.21 (10)	C9—N2—C16—S1	−9.92 (16)

### Hydrogen-bond geometry (Å, º)

*Cg*2 is the centroid of the C1–C6 ring.

**Table:**

*D*—H···*A*	*D*—H	H···*A*	*D*···*A*	*D*—H···*A*
N3—H1*N*3···S1^i^	0.866 (17)	2.664 (17)	3.4559 (12)	152.5 (15)
N3—H2*N*3···S1^ii^	0.84 (2)	2.60 (2)	3.4142 (12)	164.2 (18)
N4—H2*N*4···N1	0.881 (17)	2.209 (16)	2.6093 (15)	107.2 (13)
N4—H2*N*4···O1^ii^	0.881 (17)	2.567 (17)	3.3022 (14)	141.5 (13)
C8—H8*A*···*Cg*2^i^	0.99	2.66	3.4159 (13)	133
C17—H17*B*···*Cg*3^iii^	0.98	2.94	3.8351 (15)	152

Symmetry codes: (i) −*x*+1, −*y*+2, −*z*; (ii) −*x*+1, *y*+1/2, −*z*+1/2; (iii) −*x*+1, −*y*+1, −*z*.
